# Circadian desynchrony in early life leads to enduring autistic-like behavioral changes in adulthood

**DOI:** 10.1038/s42003-024-07131-3

**Published:** 2024-11-11

**Authors:** Abhishek Mishra, Hao Lin, Rubal Singla, Nam Le, Michael Oraebosi, Dong Liu, Ruifeng Cao

**Affiliations:** 1 Department of Biomedical Sciences, University of Minnesota Medical School, Duluth, MN 55812 USA; 2 Department of Neuroscience and Cell Biology, Robert Wood Johnson Medical School, Rutgers University, Piscataway, NJ USA; 3 Department of Neurology, Robert Wood Johnson Medical School, Rutgers University, Piscataway, NJ USA; 4 Present Address: Spencer Center for Vision Research, Department of Ophthalmology, Byers Eye Institute at Stanford University School of Medicine, Palo Alto, CA 94304 USA

**Keywords:** Circadian regulation, Autism spectrum disorders

## Abstract

Circadian rhythm regulates a variety of biological processes in almost all living organisms. Modern lifestyles, e.g. transmeridian travel, night shift, light at night, etc., frequently disrupt people’s regular sleep-wake cycles and create a misalignment (circadian desynchrony) between the natural environment and the endogenous body clock, and between different circadian oscillators within the body. The long-term consequences of circadian desynchrony on neurodevelopment and adult behavior remain elusive. Increasing clinical evidence supports a correlation between the disruption of the circadian system and neurodevelopmental disorders, such as autism spectrum disorders. Despite clinical correlations, experimental evidence is yet to establish a link between circadian disturbance in early life and adult behavioral changes. Here, using a “short day” (SD) mouse model, in which mice were exposed to an 8 h/8 h light/dark (LD) cycle mimicking a “shift work” schedule from gestation day 1 to postnatal day 21, we performed a battery of behavioral tests to assess changes in adult behaviors, including sociability, affective behaviors, stereotypy, cognition and locomotor functions. In contrast to the control mice kept in a 12 h/12 h LD cycle, the adult SD mice entrained to the 8 h/8 h LD cycle, but their free running rhythms remained normal in constant darkness. Interestingly, however, the SD mice displayed diminished sociability, a reduced preference for social novelty, excessive repetitive behaviors, and compromised cognitive functions, all of which resemble characteristics of autism-like behavioral alterations. In addition, the SD mice exhibited significant anxiety- and depressive-like behaviors and impaired motor functions. By western blotting and immunostaining analyses, hyperactivation of the mTORC1/S6K1 pathway was detected in multiple forebrain regions of SD mice. These findings underscore the enduring impact of early-life circadian disruption on neurochemical signaling and behavioral patterns into adulthood, highlighting a pivotal role for circadian regulation in neurodevelopment.

## Abstract (web summary)

Circadian desynchrony by an 8 h/8 h light/dark cycle before weaning leads to long-lasting impairments in sociability, excessive repetitive behaviors, and cognitive malfunctions, all of which resemble autism-like behavioral alterations in mice.

## Introduction

The circadian clock autonomously generates roughly 24-h rhythms in almost all animals^1^. Circadian rhythms coordinate gene expression, neurophysiological functions, and animal behavior throughout their lives^2^. During the developmental phase of the brain, the circadian clock governs essential processes like neurogenesis, cell migration, and progenitor cell differentiation^3,4^. In the mature brain, circadian rhythms regulate pivotal neuronal functions such as neuronal excitability, synaptic plasticity, memory formation, and mood^5^. The cellular circadian clock operates through a network of interdependent transcriptional-translational feedback loops. Over the years, researchers have identified nearly a dozen clock genes, expressed ubiquitously across all mammalian cells. The synchronization and expression of these clock genes in various systems are masterfully orchestrated by the suprachiasmatic nucleus (SCN) in the hypothalamus, the central circadian pacemaker. Ambient light serves as the regulator, aligning rhythms within the SCN and harmonizing the body’s internal rhythms with the external light–dark (LD) cycles^6^. It is widely acknowledged that circadian rhythms are vital for sustaining overall bodily health. Disruptive light exposure, such as nocturnal illumination, irregular work shifts, or frequent transmeridian travel, can disturb these rhythms, leading to desynchronization in multiple bodily systems. In the short term, this can manifest as symptoms akin to jet lag and various sleep disorders^7^. However, the enduring consequences of circadian disruption during developmental stages in animals remain largely uncharted territory. The molecular and cellular alterations in the adult brain following prolonged circadian disturbance during neurodevelopment have yet to be thoroughly investigated.

Autism spectrum disorders (ASDs) encompass a range of intricate neurodevelopmental conditions marked by challenges in social interaction, communication impairments, and the manifestation of repetitive or stereotypical behaviors^8^. Recent studies have spotlighted potential links between ASD etiology and disruptions in the circadian clock system^9^. Specifically, investigations have unveiled a noteworthy prevalence of clock gene polymorphisms within the ASD population, notably in genes like *Per1*, *Per2*, *Npas2*, *Nr1d1* and *Rorα/β*^10–12^. These genetic variants may underlie the core symptoms observed in individuals with ASD. Furthermore, a substantial majority of ASD children, estimated at 50–80% experience sleep-related challenges, which surpasses the prevalence in the general pediatric population^13^. Additionally, anomalies in the temporal profiles of circadian hormones like cortisol and melatonin, indicative of circadian clock function, are recurrent in ASD cases^14^. Circadian rhythms are disrupted in mouse models of ASD, such as the FXS (fragile X syndrome) or BTBR mice^15–17^. These collective findings strongly indicate a potential involvement of circadian rhythm dysregulation in the pathogenesis of ASD. This emerging understanding presents a compelling avenue for further research, with the goal of delineating targeted interventions to ameliorate the challenges faced by individuals with ASD and enhance their quality of life. Such endeavors hold promise not only for individuals with ASD but also for their families and caregivers who navigate the complexities of this condition.

The long-term effects of circadian disruption on neurodevelopment and adult behavior remain to be established. Addressing this gap could enhance our understanding of how circadian rhythms influence neurodevelopment and lead to innovative strategies for treating sleep problems common in neurodevelopmental disorders. In this study, a modified “Short Day” (SD) mouse model was developed, subjecting animals to chronic circadian disruption using an 8 h/8 h light/dark cycle from embryonic day 1 to postnatal day 21. Compared to control (CTR) mice on a standard 12 h/12 h light/dark cycle, adult SD mice exhibited striking molecular, cellular, and behavioral changes akin to ASD-like traits but intact free-running circadian rhythms. This experimental study provides direct evidence linking disruptions in the circadian cycle to ASD-like behavioral changes, thereby emphasizing the critical involvement of circadian rhythms in neurodevelopment.

## Results

### Impaired sociability and preference for social novelty in SD mice

**Fig. 1:**
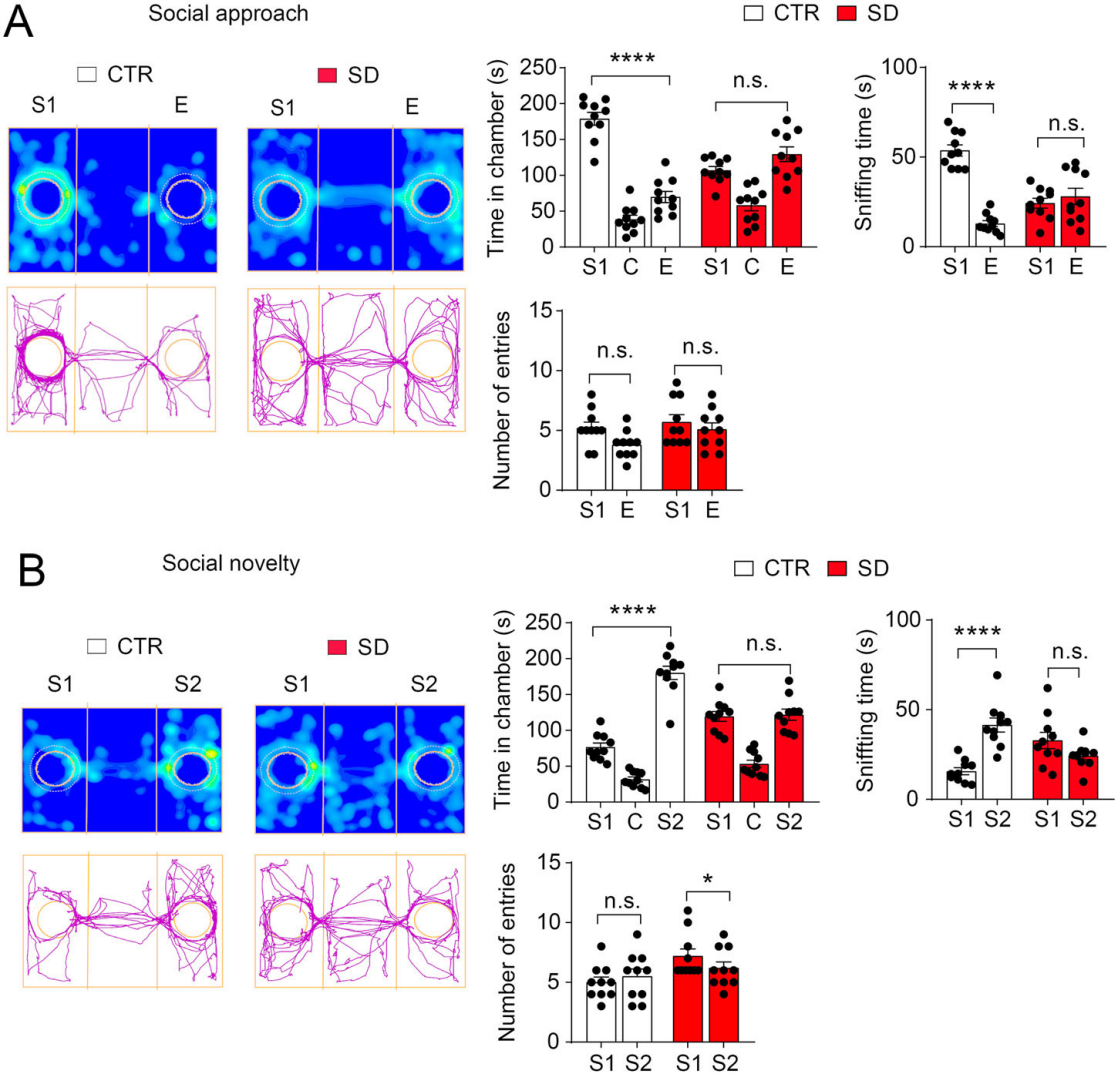
Three-chamber sociability behavior test showing deficits in the social approach and social novelty in SD. **A** SD mice displayed impairment in the social approach as equated to the CTR mice. Left (Top): Heatmap depicting the distribution of time for CTR and SD in the stranger chamber (S1), empty (E), and center (C) of the three-chamber apparatus. Left (Bottom): Track plots of the CTR and SD mice showing the movement of mice in the S1, E, and C chambers of the apparatus during the sociability phase. Right (Top): Graphical representation illustrating the time allocated in each chamber (*F*_(2, 36) chamber×SD_ = 24.92, *p* < 0.0001, *F*_(1, 18) SD_ = 2.440, *p* = 0.1357, repeated measures two-way ANOVA) and duration of time dedicated to exploring each wire cage. The CTR mice preferred the S1 over the E chamber, whereas SD mice spent a comparable amount of time in both the S1 and E chambers (CTR: S1 vs. E, *p* < 0.0001, SD: S1 vs. E, *p* = 0.1805, Bonferroni’s post hoc comparisons). Furthermore, CTR mice showed a greater duration of sniffing activity towards S1 compared to E. In contrast, SD mice spent an equal amount of time sniffing both the S1 and E cages (*F*_(1, 18) chamber×SD_ = 47.53, *p* < 0.0001, *F*_(1, 18) SD_ = 5.164, *p* = 0.0356, repeated measures two-way ANOVA, CTR: S1 vs. E, *p* < 0.0001, SD: S1 vs. E, *p* = 0.4189, Bonferroni’s post hoc comparisons). Right (Bottom): Graph depicting the frequency of entries in each chamber (*F*_(1, 36) chamber×SD_ = 0.6247, *p* = 0.4345, repeated measures two-way ANOVA). There was no discernible distinction in frequency of entries between CTR and SD mice into the S1 and E chambers (*F*_(1, 18) chamber×SD_ = 2.769, *p* = 0.1134, *F*_(1, 18) SD_ = 1.782, *p* = 0.1985, repeated measures two-way ANOVA, CTR: S1 vs. E, *p* = 0.1165, SD: S1 vs. E, *p* = 0.1801, Bonferroni’s post hoc comparisons). n = 10 mice per group. **B** The SD mice demonstrated a diminished preference for novel social stimuli. On the left side, heatmaps (above) and track plots (bottom) depict the movements of a CTR mouse and an SD mouse for the entire duration of the test. S1 denotes stranger 1, while S2 represents the novel stranger. On the right, bar graphs precisely indicate the duration spent by the mice in each chamber (*F*_(2, 36) chamber×SD_ = 21.70, *p* < 0.0001, *F*_(1, 18) SD_ = 2.869, *p* = 0.1075, repeated measures two-way ANOVA), time sniffing wire cages (*F*_(1, 18) chamber×SD_ = 25.38, *p* < 0.0001, repeated measures two-way ANOVA), and the frequency of entries into the S1 and S2 chambers (*F*_(1, 18) chamber×SD_ = 1.000, *p* = 0.3306, repeated measures two-way ANOVA). The CTR mice displayed a preference for spending more time in the chamber housing the novel stranger (S2), whereas the SD mice favored spending more time in the chamber containing the familiar stranger (S1) (CTR: S1 vs. S2, *p* < 0.0001, SD: S1 vs. S2, *p* = 0.0936, Bonferroni’s post hoc comparisons). In addition, it’s noteworthy that the CTR mice devoted more time to sniffing the S2 cage than the S1 cage, while the SD mice allocated comparable time to sniffing both the cages S1 and S2 (CTR: S1 vs. S2, *p* < 0.0001, SD: S1 vs. S2, *p* = 0.9712, Bonferroni’s post hoc comparisons). There was no disparity noted in the frequency of entries into the S1 and E chambers for the CTR mice, but the SD mice entered the S1 chamber more times than the S2 chamber (CTR: S1 vs. S2, *p* = 0.1744; SD: S1 vs. S2, *p* = 0.0111, Bonferroni’s post hoc comparisons). The sample size was *n* = 10 mice per grou*p*, with data represented as individual data points alongside the mean ± SEM. ****p < 0.0001, n.s. not significant.

We first assessed mouse circadian rhythms by their wheel-running activities. Interestingly, the SD mice were entrained to the 8 h/8 h LD cycle and exhibited intact circadian rhythms in constant darkness 28 d after being kept in the SD cycle, indicating that the SD treatment does not permanently alter mouse free-running rhythms (Fig. S1). When the SD mice were transferred to constant darkness from the SD cycle, their circadian rhythms were similar to those in CTR mice. Next, we used a three-chamber test to evaluate social behavior in both CTR and SD mice. Following a period of habituation, mice were given the opportunity to interact with a caged newer animal (S1), representing the social stimulus, or an empty cage (E), representing the non-social stimulus. Results indicated robust sociability in the CTR mice, as they spent a significantly extended amount of time in the S1 chamber than in the E chamber (*p* < 0.0001) and exhibited prolonged sniffing of the S1 cage compared to the E cage (*p* < 0.0001) (Fig. 1A). In contrast, the SD mice displayed impaired sociability, spending similar amounts of time in both the S1 and E chambers (*p* = 0.1805), as well as equivalent durations sniffing the S1 and E cages (*p* = 0.4189) (Fig. 1A). Notably, no disparities were observed in the number of entries to either chamber for either CTR (*p* = 0.1165) or SD mice (*p* = 0.1801) (Fig. 1A). Furthermore, to explore the proclivity of the animals for social novelty, a subsequent unfamiliar mouse (S2) was introduced into the previously vacant cage. As anticipated, the CTR mice demonstrated a preference for social novelty, devoting additional time to the S2 chamber than the S1 chamber (*p* < 0.0001), (Fig. 1B) and dedicating a greater duration to exploring the S2 cage in comparison to the S1 cage (*p* < 0.0001). In contrast, the SD mice exhibited a lack of preference for social novelty, as they allocated similar amounts of time to both the S1 and S2 chambers (*p* = 0.0936) and displayed comparable time sniffing the S1 and S2 cages (*p* = 0.9712) (Fig. 1B). Moreover, no significant difference was documented in the number of entries in both the chambers (S1 and S2) in the CTR (*p* = 0.1744) but the SD mice entered the S1 chamber more times than the S2 chamber (*p* = 0.0111) (Fig. 1B). Collectively, these findings signify that SD mice manifest compromised sociability and a diminished inclination towards social novelty, aligning with the established characteristic of impaired social interactions in neurodevelopmental disorders such as ASD.

### Excessive repetitive behavior in SD mice

**Fig. 2:**
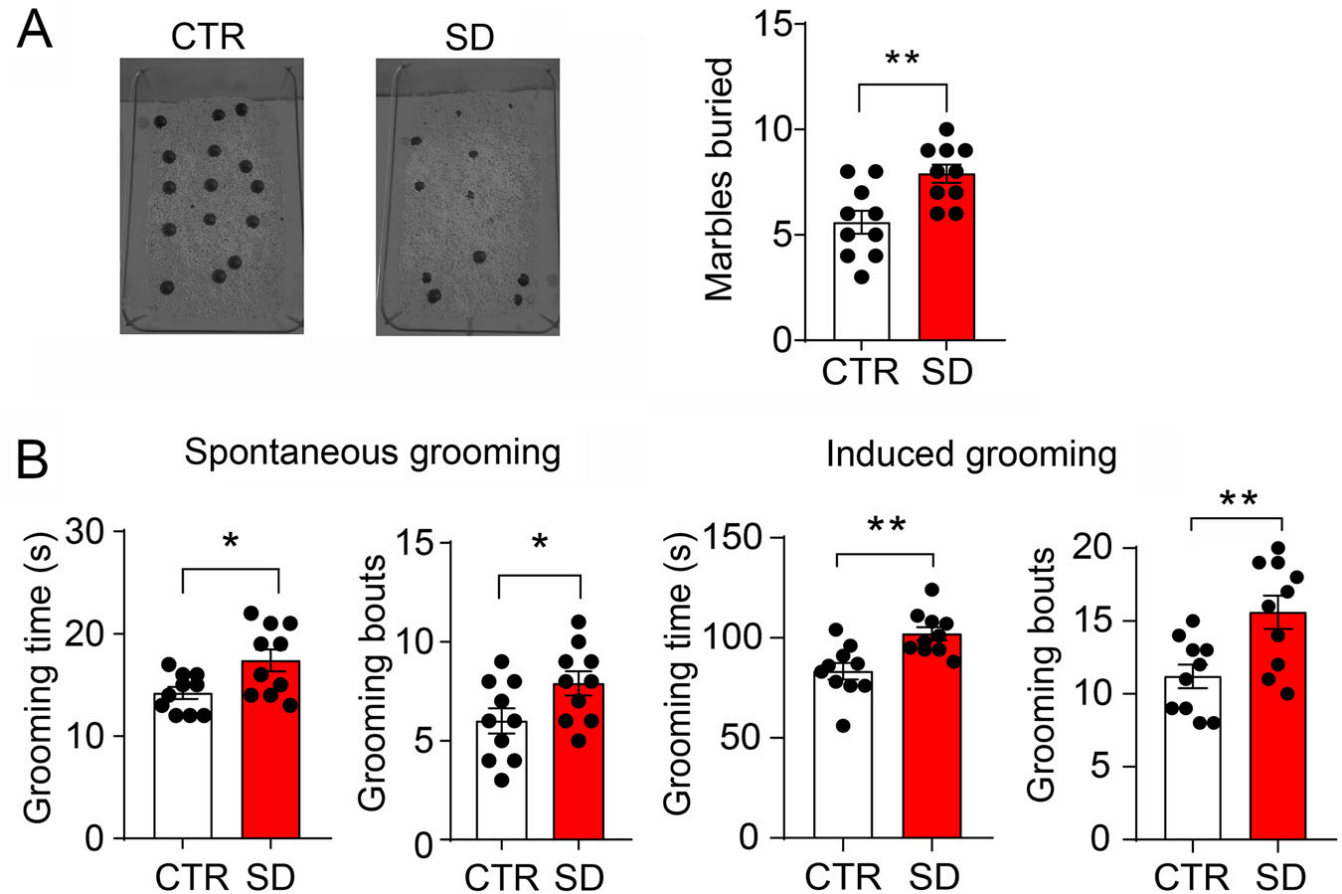
SD mice demonstrated excessive repetitive and stereotypical behaviors. **A** In the marble burying test, images exemplify marble-burying behavior in both a CTR and an SD mouse. A bar graph presents the number of buried marbles, indicating that SD mice buried a higher quantity compared to CTR mice (*t*_(18)_ = 3.316, *p* = 0.0038, Student’s *t*-test). *n* = 10 mice per group. **B** During the examination of mouse grooming patterns in the spontaneous grooming test, bar graphs illustrate grooming bouts and grooming time. It indicated significant differences for both groups in grooming bouts (*t*_(18)_ = 2.172, *p* = 0.0435, Student’s *t*-test) and grooming time (*t*_(18)_ = 2.623, *p* = 0.0173, Student’s *t*-test). In the induced grooming test, conducted by stimulating mice with a gentle water puff to elicit grooming behavior, bar graphs illustrate the grooming bouts as well as the total time spent on grooming. The results unveiled noteworthy distinctions for both instances of grooming bouts (*t*_(18)_ = 3.129, *p* = 0.0058, Student’s *t*-test) and total grooming time (*t*_(18)_ = 3.502, *p* = 0.0025, Student’s *t*-test), with SD mice exhibiting heightened values compared to CTR mice. *n* = 10 mice per group. The data are displayed as individual data points alongside the mean ± SEM. *p < 0.05, **p < 0.01, ns, not significant.

To assess stereotypical and repetitive behaviors in mice, we conducted evaluations focusing on marble burying and grooming tendencies in both CTR and SD mice. Strikingly, SD mice exhibited a significantly higher rate of marble burial compared to their CTR counterparts (*p* = 0.0038, Fig. 2A). Additionally, the SD mice demonstrated an increased frequency of spontaneous grooming bouts (*p* = 0.0435) and extended the duration of grooming activities (*p* = 0.0173) in comparison to the CTR mice (Fig. 2B). Furthermore, in the water spray-induced grooming test, the SD mice displayed a notable escalation in both the digits of grooming bouts (*p* = 0.0058) plus the total period spent in grooming (*p* = 0.0025) when contrasted with the CTR mice. These findings collectively underscore the substantial presence of repetitive behaviors in SD mice, further mirroring core features observed in ASD.

### Cognitive impairments in SD mice

**Fig. 3:**
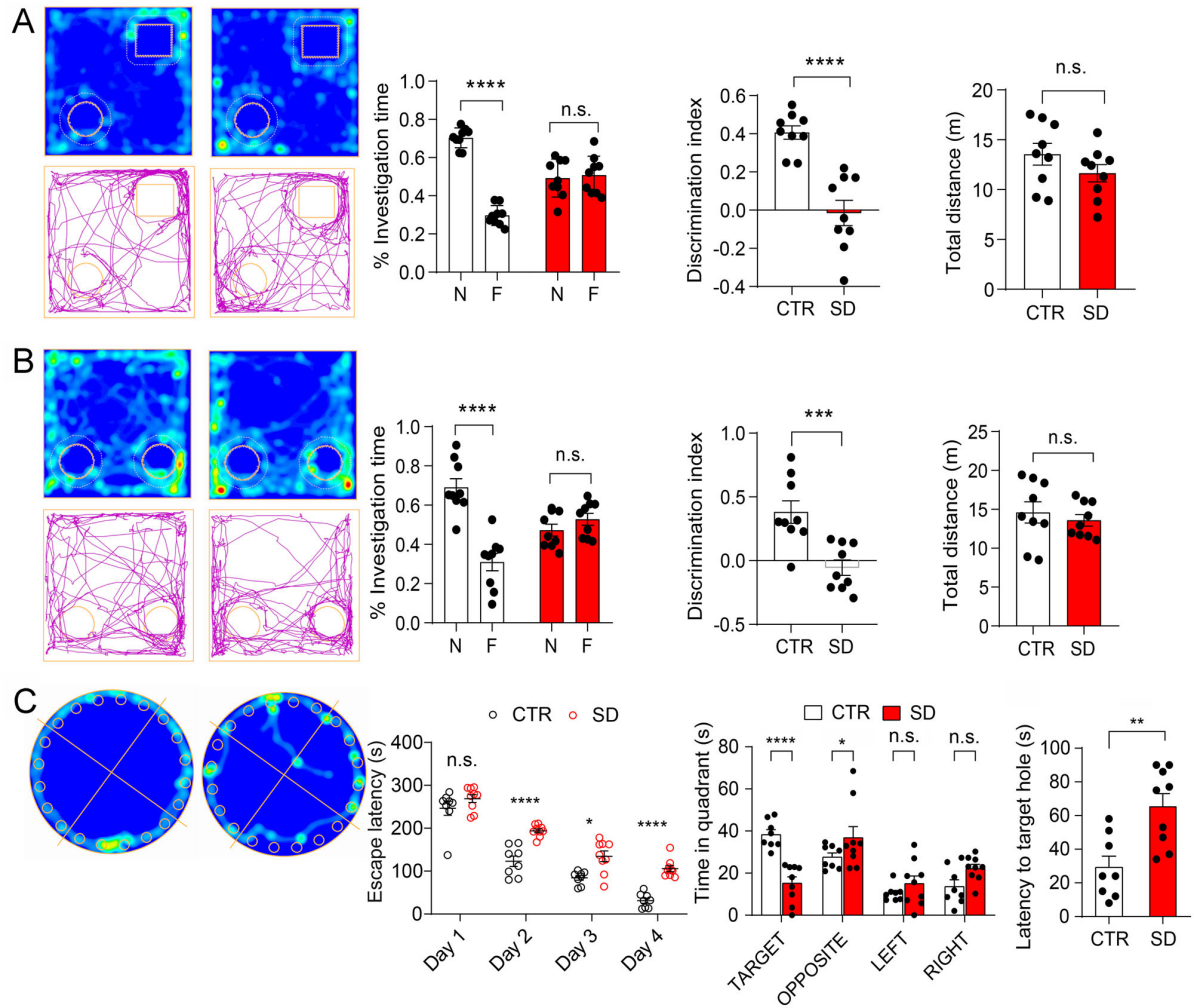
Impaired cognitive functions in SD mice. **A** In the novel object recognition (NOR) test, heat maps depict the duration spent at specific locations by both CTR and SD mice on the left side. On the right side, a bar graph demonstrates the time dedicated to exploring novel or familiar objects, along with the Discrimination Index (DI). The DI was calculated as (Time _novel_−Time _familiar_)/(Time _novel_ + Time _familiar_. Bar graphs showing the time investigating novel and familiar objects (*F*_(1,16) objects×SD_ = 31.75, *p* < 0.0001, repeated measures two-way ANOVA). The CTR mice showed preference for the novel object (N) than familiar (F), while SD spent similar time with novel and familiar object (CTR: N vs. F, *p* < 0.0001, SD: N vs. F, *p* = 0.7793, Bonferroni’s post hoc comparisons). The DI of CTR animals was also higher in comparison to SD mice (*t*_(*16)*_ = 5.635, *p* = 0.0001, Student’s *t*-test). The total dis*t*ance traveled by CTR was similar to SD mice (*t*_*(16)*_ = 1.366, *p* = 0.1907, Student’s *t*-test). *n* = 9 mice in CTR and 9 mice in SD. **B** In the Object Location Memory (OLM) test, illustrative heat maps depict the duration of time at each location by both CTR and SD mice on the left. On the right, a bar graph illustrates the time dedicated to exploring novel or familiar objects and calculating the DI. The DI was calculated as (Time _novel_−Time _familiar_)/(Time _novel_ + Time _familiar_). Bar graphs display the time investigating novel and familiar locations (*F*_(1,16) location×SD_ = 15.56, *p* = 0.0009, repeated measures two-way ANOVA). It’s noteworthy that CTR mice spent a greater amount of time with a novel object (N) compared to the familiar (F) location, while SD mice dedicated a comparable amount of time with both novel and familiar locations (CTR: N vs. F, *p* = 0.0001, SD: N vs. F, *p* = 0.4786, Bonferroni’s post hoc comparisons). Additionally, the DI was higher in CTR animals compared to SD mice (*t*_(16)_ = 4.069, *p* = 0.0009, Student’s *t*-test). The total distance traveled by CTR was similar to SD mice (t(16) = 0.6510, p = 0.5243, Student’s t-test). *n* = 9 mice in CTR and 9 mice in SD. **C** Barnes Maze test. Left: Representative heat plot indicates time spent by CTR and SD mice during the test. Middle: Line graph showing the time taken to escape on each day (Day 1–4) of the acquisition phase. The escape latency of both the CTR and SD mice decreased over each day. However, the SD mice took more time to escape from the maze during each day of the acquisition phase compared to the CTR mice (*F*_(1,15) SD_ = 79.29, *p* < 0.0001, *F*_(3,45) day_ = 128.5, *p* < 0.001, *F*_(3,45) SD×day_ = 2.772, *p* = 0.0523, repeated measures two-way ANOVA; CTR vs SD Day1: *p* = 0.1096; Day2: *p* < 0.0001; Day3: *p* = 0.0006; Day4: *p* < 0.0001, Bonferroni’s post hoc comparisons). Right: Bar graph showing the time spent by SD and CTR mice in different quadrants (*F*_(1,15) SD_ = 0.8816, *p* = 0.3626, *F*_(3,45) quadrant_ = 11.53, *p* < 0.0001, *F*_(3,45) SD×quadrant_ = 8.963, *p* < 0.0001, repeated measures two-way ANOVA) and latency to reach the target hole (*t*_(15)_ = 3.547, *p* = 0.0029 Student’s *t*-test). The SD mice spent a significantly reduced amount of time in the target quadrant (*p* < 0.0001) and a greater amount of time in the opposite (*p* = 0.1339), left (*p* = 0.2548) and right (*p* = 0.0427) quadrants in comparison to CTR mice (repeated measures two-way ANOVA). The CTR mice spent significantly more time in the target quadrant (target vs. opposite, *p* = 0.0588, target vs. left, *p* = 0.0003, target vs. right, *p* = 0.0089), whereas the SD mice spent similar time in four quadrants (target vs opposite, *p* = 0.1604, target vs. left, *p* > 0.9999, target vs. right, *p* = 4211). The data was depicted as individual values, accompanied by the mean ± SEM. **p < 0.01, ***p < 0.001, ****p < 0.0001, n.s. not significant. *n* = 8 mice in CTR and 9 mice in SD. A mouse in CTR spent more time in the opposite quadrant than the target quadrant, and its data were removed from all analyses of the Barnes Maze test.

Cognitive impairments are frequently associated with neurodevelopmental disorders such as ASD^18^. To assess the cognitive functions in SD mice, we conducted the Novel Object Recognition (NOR) memory test, which evaluates non-spatial memory related to object identity (Fig. 3A). The CTR mice exhibited a strong preference for the novel object (N) over the familiar object (F) (*p* < 0.0001). In contrast, SD mice spent nearly equal amounts of time with both the novel and familiar objects (*p* = 0.7793). Further, analysis of the Discrimination Index (DI) revealed a significantly higher value in CTR mice (*p* < 0.0001) compared to SD mice. Interestingly, there was no significant difference observed in the distance traveled by the CTR and SD mice (*p* = 0.1907). Next, we conducted an Object Location Memory (OLM) test to assess spatial memory, a function primarily reliant on the hippocampus. Among the CTR mice, a substantial preference was observed for the object positioned in a novel location 24 h after training (*p* = 0.0001) (Fig. 3B). Conversely, the SD mice exhibited a comparable preference for both the novel and familiar-located objects (*p* = 0.4786). Furthermore, a significant disparity in the Discrimination Index (DI) was noted between the CTR and SD mice (*p* = 0.0009). Interestingly, there was no substantial distinction in the distance traveled by either CTR or SD mice (*p* = 0.5243). These collective findings indicate an impairment in long-term spatial memory among SD mice.

To complement the OLM test, we next performed the Barnes maze test (Fig. 3C), which is used to assess spatial learning and memory. CTR mice exhibited significant learning progress in locating the escape point. This was evident in their consistent reduction of escape latency over the four training days in the acquisition phase, indicating learning and memory retention abilities. Throughout the acquisition phase, both the CTR and SD mice demonstrated daily improvements in proficiency in escape latency (*F*_(3,45) day_ = 128.5, *p* < 0.001). However, during the acquisition phase, the CTR mice had lower escape latencies over each day as compared to the SD mice (Day 1: *p* = 0.1096; Day 2: *p* < 0.0001; Day 3: *p* = 0.0006; Day 4: *p* < 0.0001), underscoring impaired spatial learning capabilities in SD mice. In the subsequent probe test, the SD mice showed a significantly longer latency in reaching the target hole compared to the CTR mice (*p* = 0.0029), indicating impaired memory in SD mice. Additionally, we analyzed the time spent in the target, opposite, left, and right quadrants by the CTR and SD mice during the probe test. The time spent was notably higher in the target quadrant (*p* < 0.0001) and lower in the right quadrant (*p* = 0.0427) in the CTR mice compared to the SD mice. However, no significant differences were observed in the other zones (*p* = 0.1339 for the opposite, *p* = 0.2548 for the left) between the CTR and the SD mice. Together, these results demonstrate impaired spatial learning and memory in SD mice.

### Anxiety-like behavior and motor learning impairments in SD Mice

**Fig. 4:**
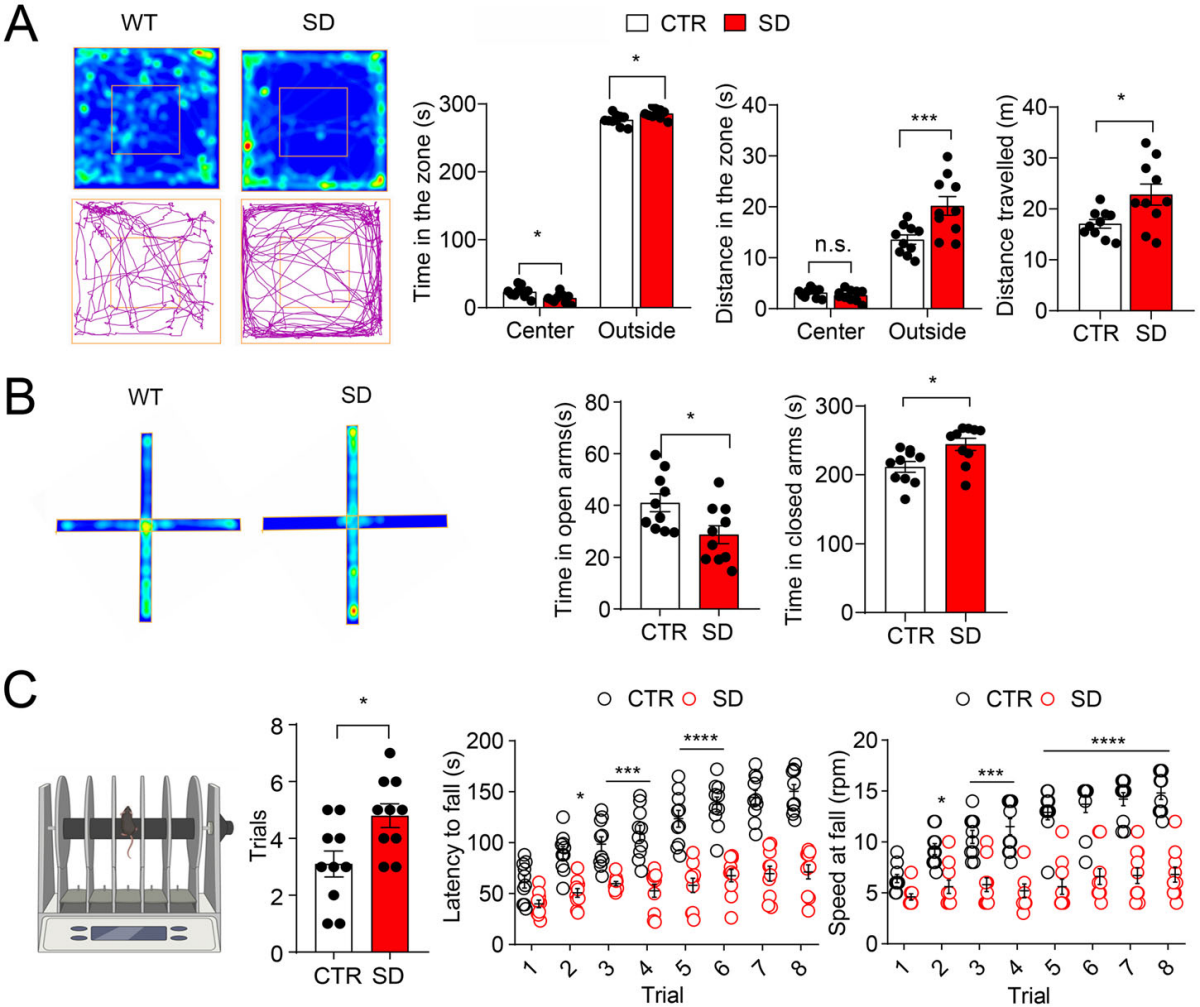
Increased anxiety-like behavior in SD mice. **A** Open field test includes illustrative heat maps (above) and track plots (below) of CTR and SD mice. The left side illustrates the time spent and distance covered throughout the test. Bar graphs on the right side display the time and distance covered within the center zone and outer zones, as well as the total distance traveled. Notably, SD mice spent less time in the center and outside zones compared to CTR mice (*F*_(1,18) ZoneXSD_ = 7.478, *p* = 0.0136, *F*_(1,18) Zone_ = 6135, *p* < 0.0001, two-way ANOVA. Center: CTR vs SD, *p* = 0.0193; Outside: CTR vs. SD, *p* = 0.0193, Bonferroni’s post hoc comparisons). The SD mice traversed a greater distance in the outer zone and a comparable distance in the central zone in comparison to the CTR mice (*F*_(1,18) Zone X SD_ = 13.05, *p* = 0.0020, *F*_(1,18) Zone_ = 201.5, *p* < 0.0001, two-way ANOVA; Center: CTR vs. SD, *p* > 0.9999; Outside: CTR vs. SD, *p* = 0.0002, Bonferroni’s post hoc comparisons). Moreover, the total distance traveled by SD mice exceeded that of CTR mice (*t*_*(*18)_ = 2.587, *p* = 0.0186, Studen*t*_*’*_s *t*-test). *n* = 10 mice per group. **B** Elevated plus maze test. Left: representative image of heat (time) and track (distance) plots. It is noteworthy that the SD mice had reduced duration in open arms as compared to CTR mice (*t*_(18)_ = 2.489, *p* = 0.0228, and more time in closed arms as compared to CTR mice *t*_(18)_ = 2.770, *p* = 0.0126. Studen*t*’s *t*-test). *n* = 10 mice per group. **C** The rotarod test involved pretraining mice to remain on the rotating rod at 4 rpm for a minimum of 1 min prior to the trials. The left diagram illustrates the apparatus used for testing. The center bar graph illustrates that SD mice required a higher number of pretrials compared to CTR mice (*t*_(18)_ = 2.746, *p* = 0.0133, Studen*t*’s *t*-test). The right line graph demonstrates the speed at which mice fell and the latency to fall. Notably, the performance of CTR mice improved over eight trials, whereas SD mice did not show improvement (*F*_(1,18) SD_ = 183.4, *p* < 0.0001, *F*_(7,126) trialxSD_ = 5.573, *p* < 0.0001, *F*_(7,126) trial_ = 22.22, *p* < 0.0001, repeated measures two-way ANOVA). Additionally, SD mice descended within a shorter latency compared to the CTR mice (trial 1: *p* = 0.0187; trial 2: *p* < 0.0001; trials 3–8: *p* < 0.0001; Bonferroni’s *p*ost hoc comparisons) and exhibited markedly reduced s*p*eed at fall (*F*_(1,18) SD_ = 161.3, *p* < 0.0001, *F*_(7,126) trialxSD_ = 6.036, *p* < 0.0001, *F*_(7,126) trial_ = 15.89, *p* < 0.0001, repeated measures two-way ANOVA; CTR vs. SD: trial 1: *p* = 0.0602; trial 2: *p* = 0.0002; trials 3–8: *p* < 0.0001, Bonferroni’s post hoc comparisons) in comparison to the CTR mice. *n* = 10 mice per grou*p*. Data represented as individual data points, as well as the mean ± SEM. *p < 0.05, ***p < 0.001, n.s. not significant.

Anxiety frequently co-occurs with neurodevelopmental disorders. In this current study, we further investigated anxiety- and depressive-like behaviors in SD mice utilizing open field and elevated plus maze tests. During the open field test, the SD animals exhibited decreased time in the center region (*p* = 0.0193) but extended time in the outside region (*p* = 0.0193) relative to the CTR mice (Fig. 4A). Furthermore, the SD mice traveled a greater distance in the outside zone (*p* = 0.0002) and overall (*p* = 0.0186) compared to the CTR mice, signaling heightened anxiety-like behaviors. Additionally, the elevated plus maze results indicate that SD mice spent less time in the open arm (*p* = 0.0228) and more time in the closed arm (*p* = 0.0126) compared to their CTR counterparts (Fig. 4B), further indicating an anxiety-like condition in SD mice. Given the consistent reports of impaired motor skills in ASD, we conducted a rotarod test to assess motor learning and coordination in SD mice. Our findings revealed that SD mice required a greater number of pretraining trials compared to CTR mice (*p* = 0.0133). Moreover, while motor performance significantly improved in the CTR mice (*p* < 0.0001) over the eight trials, the SD mice exhibited significantly shorter latencies to fall in Trial 1 (*p* = 0.0187), Trials 2–8 (*p* < 0.0001) in contrast to the CTR animals (Fig. 4C). Additionally, the SD animals fell at considerably reduced rate of rotation in Trial 2 (*p* = 0.0002), and 3–8 (*p* < 0.0001) than their CTR counterparts. There was no difference in Trial 1 (*p* = 0.0602). These results collectively highlight substantial deficits in motor learning and coordination in SD mice.

### Depressive-like behaviors in SD mice

**Fig. 5:**
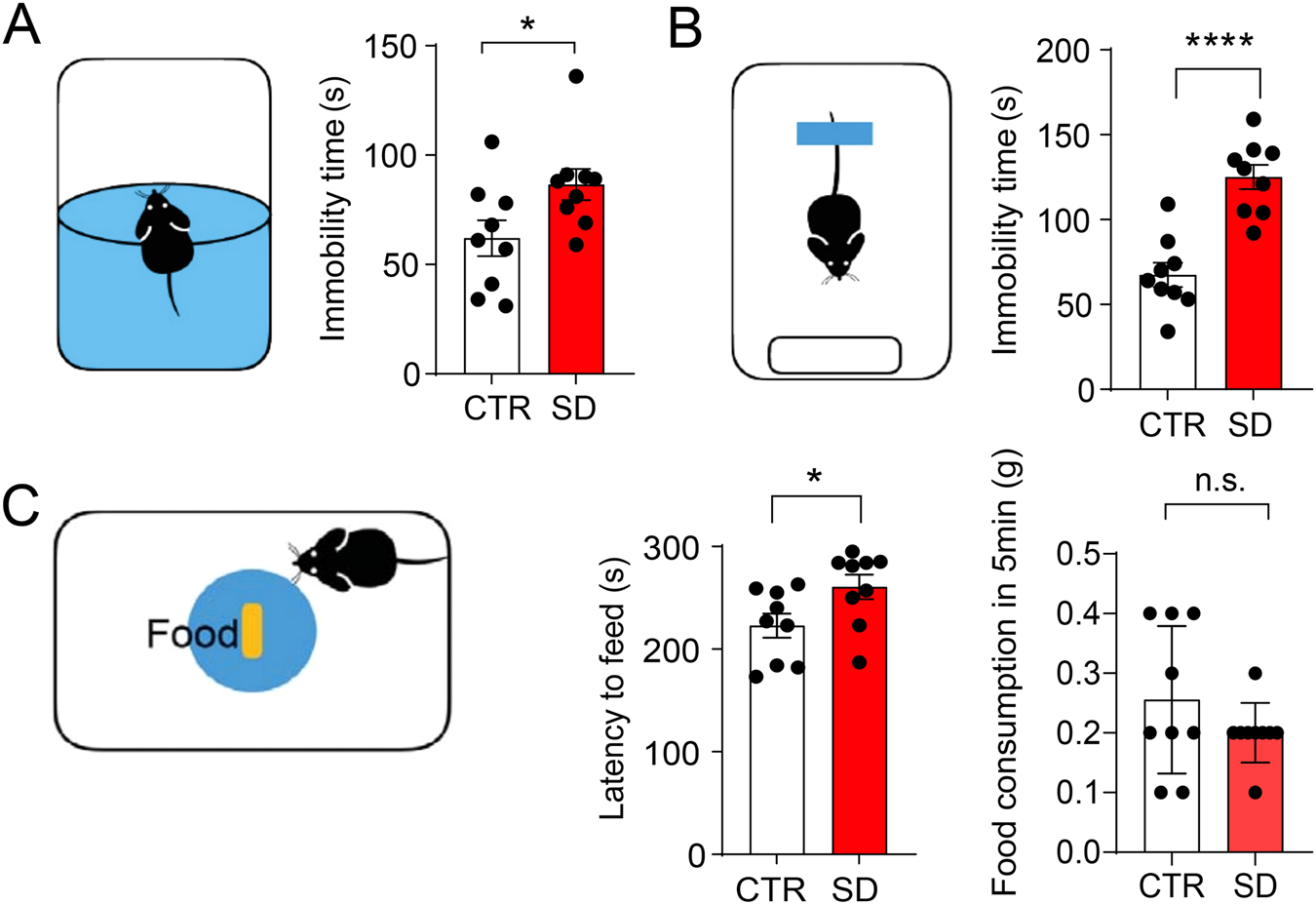
SD mice exhibit depressive-like behavior. **A** Forced swim test (FST) Left: Schematic representation of FST. Right: Bar graph indicates the immobility time. Note that immobility time was longer in SD mice in comparison to CTR mice. (*t*_(16)_ = 2.255, *p* = 0.0385, Student’s *t*-test) *n* = 9 mice per group. **B** Tail suspension test (TST). Left: Schematic representation of TST. Right: Bar graph indicates the immobility time. Note that immobility time was longer in SD mice as compared to CTR mice *(t*_(16)_ = 5.705, *p* < 0.0001, Student’s *t*-test). *n* = 9 mice per group. **C** Novelty-suppressed feeding. Left: Schematic representation of NSF. Right: Bar graph indicates latency to feed and home cage consumption of food. Note that latency to feed was higher in SD mice as compared to the wild-type group (*t*_(16)_ = 2.257, *p* = 0.0384, Student’s *t*-test). Home cage consumption of food was lower in SD mice as compared to CTR mice but not statistically significant (*t*_(16)_ = 1.250, *p* = 0.2293. Student’s *t*-test) was utilized, *n* = 9 mice per group. Data are depicted as individual data points, accompanied by the mean ± SEM. *p < 0.05, ****p < 0.0001, n.s. not significant.

Depression is also a common comorbidity associated with neurodevelopmental disorders, we assessed depressive-like behaviors in SD mice by forced swim, tail suspension and novelty suppressed feeding tests. Both the forced swim and tail suspension tests measure the amount of immobility time. We found that in the forced swim test, the SD mice exhibited a longer immobility time compared to CTR mice (*p* = 0.0385) (Fig. 5A). Similarly, in the tail suspension test, the SD mice showed a significantly higher immobility time compared to the CTR mice (*p* < 0.0001) (Fig. 5B). To further confirm these depressive-like behaviors, we conducted the novelty suppressed feeding test. Interestingly, we found that the SD mice took longer to start feeding compared to the CTR mice (*p* = 0.0384). In their home cages, the SD mice consumed less food than the CTR mice, although this difference was not statistically significant (*p* = 0.2293) (Fig. 5C). Taken together, these results provide strong evidence that SD mice exhibit behaviors indicative of depressive-like states.

### Hyperactivation of the mTORC1 signaling pathway in the brain of SD mice

**Fig. 6:**
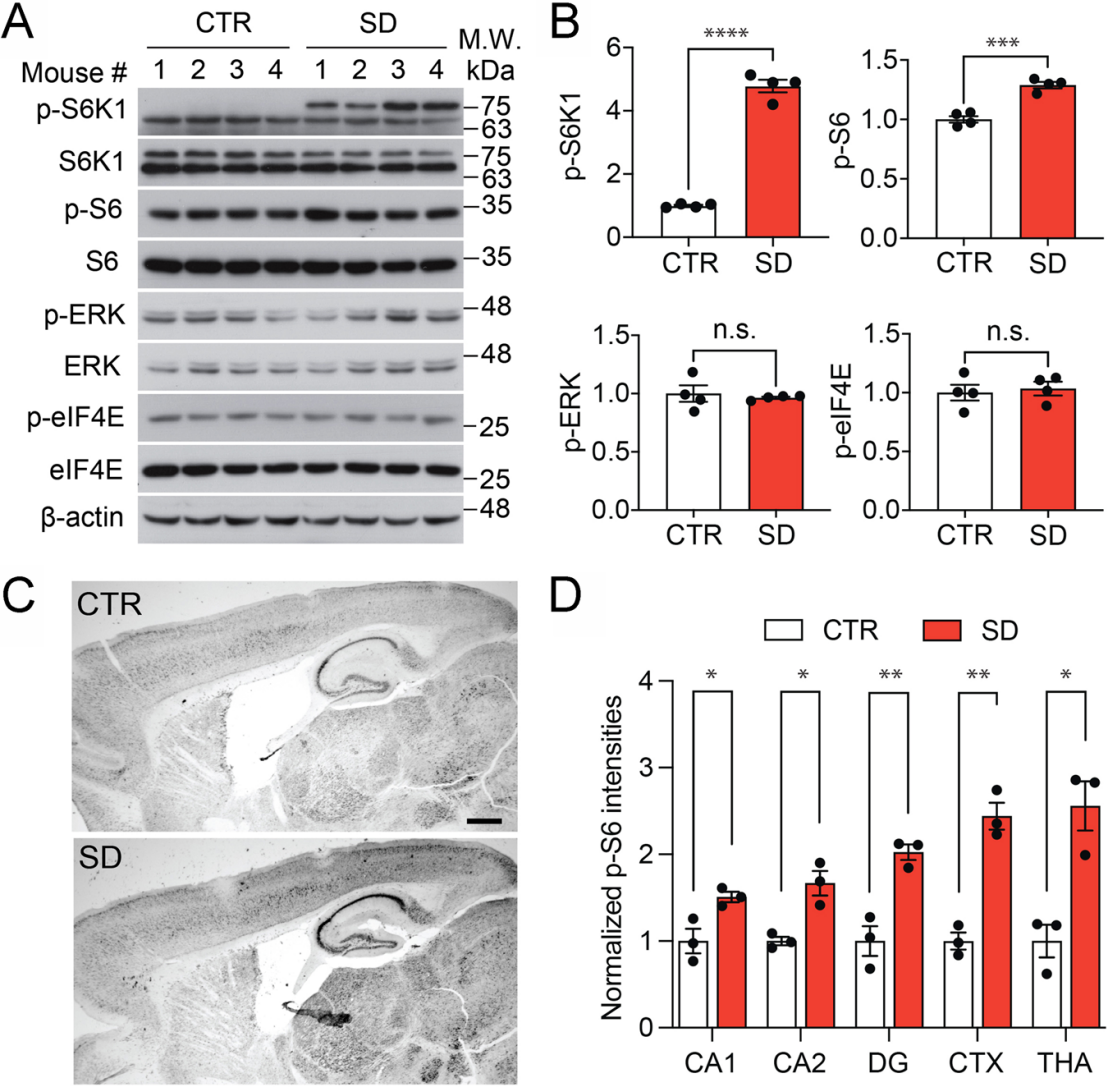
The mTORC1 signaling pathway is hyperactivated in SD mice. **A** Representative western blots showed the levels of p-S6K1, p-S6, p-ERK, and p-elF4E in the forebrain of control (CTR) and short-day cycle (SD) mice. ${{{\rm{\beta }}}}$-Actin was detected as the loading control. *n* = 4 mice per group. Also see uncropped western blotting images in Fig. S2. **B** The bar graphs of blot intensity quantification were displayed for the p-S6K1, p-S6, p-ERK, and p-elF4E relative expression. The blot intensity levels were normalized to the levels in CTR mice. It was demonstrated that the expression of p-S6K1 (*t*_(6)_ = 18.61, *p* < 0.0001, Student’s *t*-test), and p-S6 (*t*_(6)_ = 7.524, *p* = 0.0003, Student’s *t*-test) significantly increased in SD mice compared to CTR mice. Meanwhile, the expression of p-ERK (*t*_(6)_ = 0.5152, *p* = 0.6248, unpaired t-test) and p-elF4E (*t*_(6)_ = 0.3899, *p* = 0.7100, unpaired *t*-test) were similar in both CTR and SD mice. *n* = 4 mice per group. **C** Representative microscopic images of immunostaining for p-S6 in the mouse forebrain. Scale bar: 500 µm. **D** The bar graph indicates quantification of p-S6 staining intensities in the CA1, CA2, and dentate gyrus (DG) of the hippocampus, as well as in the cortex (CTX) and the thalamus (THA). The intensity levels were normalized to those in CTR mice. The results indicated a significant increase in the levels of p-S6 was in CA1 (*t*_(4)_ = 3.303, *p* = 0.0299), CA2(*t*_(4)_ = 4.465, *p* = 0.0111), DG(*t*_(4)_ = 5.283, *p* = 0.0062), CTX(*t*_(4)_ = 7.835, *p* = 0.0014) and THA(*t*_(4)_ = 4.570, *p* = 0.0103) brain regions. *n* = 3 mice per group. *p < 0.05, **p < 0.01, ***p < 0.001, n.s., not significant by Student’s *t*-test.

Altered mTOR signaling cascade has been suggested as a triggering factor in the onset of idiopathic autism^19^. Our previous work indicates that the activities of the mTOR and MAPK pathways are increased in the hippocampi of mouse offspring kept permanently in the SD cycle^6^. To determine the activities of the mTOR and MAPK pathways in the current SD mouse model, we first performed western blotting using forebrain lysates. We found elevated levels of p-S6K1(*p* < 0.0001) and p-S6 (*p* = 0.0003) in the SD brain as compared to the CTR brain, the pivotal downstream effectors of the mTORC1 signaling pathway (Figs. 6A, B, and S2), indicating hyperactivation of the mTORC1 pathway in SD mice. In contrast, the levels of p-eIF4E (*p* = 0.7100) and p-ERK (*p* = 0.6248) were not changed (Figs. 6A, B, and S2), indicating that the activities of the MAPK/MNK pathways remained normal in SD mice. To confirm the western data, we performed immunostaining for p-S6 in the forebrain. Interestingly, we found the levels of p-S6 were increased in various brain regions, including the hippocampal CA1 (*p* = 0.0299), CA2 (*p* = 0.0111), and the dentate gyrus (DG) (*p* = 0.0062) areas, as well as the cortex (*p* = 0.0014) and thalamus (*p* = 0.0103) (Fig. 6C and D), which are consistent with the western data and indicate widespread mTORC1 hyperactivation in the brain of SD mice.

## Discussion

Clinical evidence has increasingly associated circadian dysfunction with neurodevelopmental disorders such as ASD^13,20^. However, experimental proof is necessary to test a causal link between circadian rhythm disruption and impaired neurodevelopment and animal behavior in adulthood. Animal models are frequently employed to explore the causal impact of genetic and environmental factors on neurodevelopmental disorders. This study utilized a modified SD mouse model, which was kept in the circadian rhythm disruptive 8 h/8 h light/dark cycle from E1 to P21. We find that circadian rhythm disruption during early life results in significant changes in adult behaviors, including impaired sociability, excessive repetitive behaviors, and impaired cognitive functions, all of which resemble autistic-like behavioral changes. These behavioral changes are associated with hyperactivation of the mTORC1 pathway in the brain. These results indicate that circadian disruption in early life could impair neurodevelopment and lead to long-lasting adverse effects on mental health.

In the technology-driven world, modern work and lifestyle (light at night, artificial blue light emission from electronics, irregular sleep schedules, and shift work, etc.) often involve irregular light exposure, which can significantly disrupt our circadian rhythms. The 8 h/8 h light/dark cycle was designed to be a simplified way to disrupt mouse circadian rhythms with light. The 8 h light phase and 8 h dark phase alternates daily so it is easily predictable when the light is on and when mice can be checked, which facilitates animal husbandry for such a long-term experiment. The experimental setup does not mimic photoperiodic/seasonal variations in day length. The current study aimed to uncover long-term neurodevelopmental consequences of circadian disruption by rhythm-disruptive light exposure.

In our previous study, we reported similar behavioral changes in an SD mouse model, which were kept in the 8 h/8 h light/dark cycle throughout life^6^. The current modified SD model is maintained under the standard 12 h/12 h LD cycle after weaning. In the previous SD model, we observed significant hyperactivation of both mTORC1 and ERK MAPK pathways in the forebrain of SD mice^6^. In the current model, we only find significant changes in the mTORC1 pathway, suggesting that the aberrant activities of the mTORC1 pathway could underlie behavioral changes in the modified SD mice. In addition, comparing the two models, circadian disruption in early life seems to be sufficient to cause autistic-like behavioral changes. This is consistent with the fact that ASD patients are usually diagnosed in early childhood between the ages of 18 and 24 months^21^, indicating that most pathological changes caused by circadian disruption are already formed before weaning and can last into adulthood.

Interestingly, the SD mice exhibited intact circadian rhythms in constant darkness, indicating that circadian parameters will resume to the normal level once the SD light cycle is removed (Fig. S1). In the wheel-running experiment using Cohort 3, the SD treatment was lengthened by 28 days, comparing to mice in Cohorts 1 and 2, as the wheel-running tests could not be performed using pre-weaning mice. Even after the prolonged SD treatment, the free-running rhythms remained normal in SD mice, indicating that the SD cycle cannot permanently alter the circadian clock function. After weaning, the Cohorts 1 and 2 of CTR and SD mice were maintained in a standard 12 h/12 h Light/Dark cycle. All behavioral tests were performed in the light phase from ZT2-5. Thus, the behavioral changes in SD mice are caused by neurodevelopmental changes rather than time-of-day effects.

Our earlier investigation concentrated on the hippocampus for molecular and cellular examination, given its significance in cognitive functions and its implication in the pathogenesis of autism^22,23^. The hippocampus, crucial for long-term memory, exhibits circadian rhythmicity in neuronal activities. This corresponds to the daily fluctuations in our learning and memory capabilities^24^. Circadian rhythms extend their influence on hippocampal long-term potentiation at CA1 synapses, firing rates of CA1 place cells, and the processes of memory acquisition and recall. Disruptions in essential circadian genes like *Per1*, *Cry1/2*, *Clock*, and *Arntl* (*Bmal1*) have been linked to deficits in hippocampal-dependent learning tasks in mice^22,23,25^. Additionally, the hippocampus is among the extra-suprachiasmatic nucleus (extra-SCN) regions demonstrating strong circadian oscillations^22,26,27^. Building on this literature, we conducted NOR, OLM, and Barnes Maze tests to assess memory impairments in SD mice. Upon careful analysis of the results, NOR findings indicate that SD mice exhibited impaired memory, as evidenced by their inability to recognize the novel object. They spent a similar amount of time with both the novel and familiar objects, resulting in a lower discrimination index. Additionally, when assessing spatial memory through the OLM test, SD mice failed to recognize the novel location. Unlike CTR mice, which spent more time exploring the novel location, SD mice displayed a lack of discrimination between the novel and familiar locations. These results suggest long-term memory impairment in SD mice. However, it is worth mentioning that the SD model is a model of global circadian disruption. Light disrupts the SCN rhythms, and in turn, the rhythms in the peripheral brain regions and peripheral organs are all disrupted. Future work is needed to pinpoint the brain regions most responsible for the behavioral changes.

Scientific investigations into ASD and the concurrent presence of anxiety/depression during childhood and adolescence consistently highlight a higher prevalence of symptoms in individuals with ASD compared to their typically developing counterparts^28^. The reported rates for anxiety and major depressive disorder in this cohort range between 17–70% and 42–56%, respectively. Kuusikko et al. further observed an elevation in parent-reported anxiety/depression symptoms among 8–15-year-olds diagnosed with high-functioning autism or Asperger syndrome^29^. Additionally, adolescents aged 12 and above exhibited heightened self-reported symptoms of social anxiety^30^. In our study, we explored anxiety-like behaviors in mice through the elevated plus maze and open field test. Interestingly, we found that SD mice spent more time in the closed arms and less time in the open arms compared to CTR mice, indicative of anxious behavior. This was further supported by the open field test, where SD mice spent more time in the outer zone and less time in the center zone, covering more distance in the outer zone. Collectively, these results suggest that SD mice exhibit anxiety-like behaviors. Furthermore, we assessed depressive behavior using the forced swim test and tail suspension test, observing that SD mice displayed longer immobility times compared to CTR mice. This was corroborated by the novelty-suppressed feeding test, where SD mice exhibited a longer latency to feed compared to CTR mice, indicating depressive-like behavior. These findings are consistent with previous studies reporting increased stress and anxiety-like behavior in various “jet lag” models^31–34^.

During the mouse gestational and newborn period before eye-opening, the offspring rhythms are largely entrained by rhythmic signals from the dam^35^. As adult C57BL/6J mice cannot entrain to extremely short light cycles such as the 8 h/8 h LD cycle, the maternal circadian rhythms are disrupted by the SD cycle, which supposedly disrupts the rhythms of the embryos and fetuses. One limitation of the current SD model, however, is that the effects of parental stress caused by circadian disruption and circadian disruption effects on the offspring cannot be separated. As the SD cycle was applied to the breeder mice, the SD offspring inevitably are subject to the impact of the SD cycles on the sires (for example, increased irritability due to repeated sleep disruption), in addition to the direct effect of circadian disruption on themselves. Thus, the effects we observed can be a combination of parental and offspring effects caused by the SD cycle.

In summary, the current study establishes a modified SD model, which may be used as a novel model to study the environmental disruption of circadian rhythms on neurodevelopment and animal behavioral changes in adulthood. The findings demonstrate long-lasting adverse consequences of early life circadian disruption for adult behaviors. Modern lifestyle, e.g. transmeridian travel, night shift, and light at night, frequently disrupts people’s regular daily rhythms^36^. The results highlight the potential impact of circadian disruptions in children and adolescents on their adult behavior.

## Methods

### Animals

**Fig. 7:**
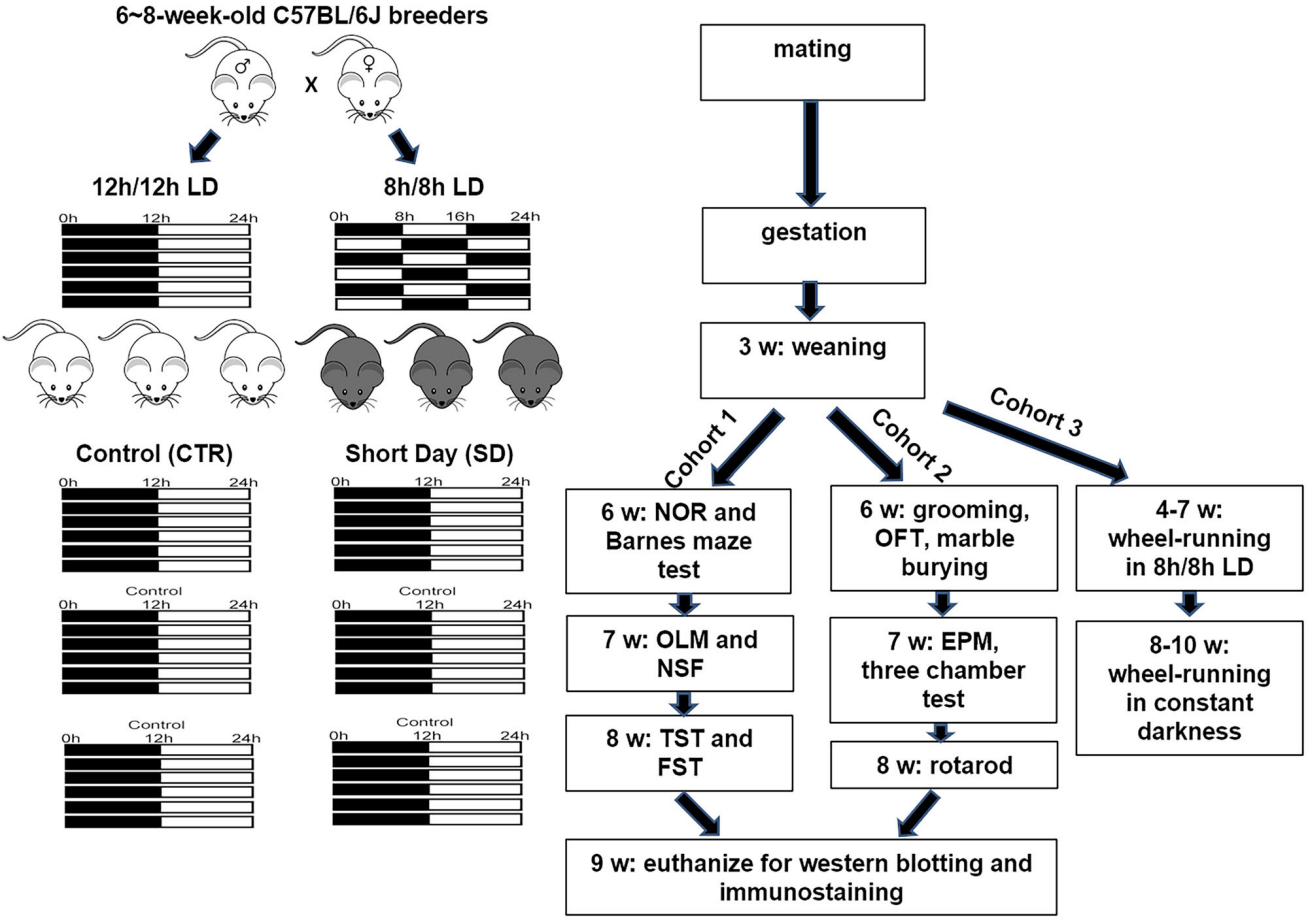
Experimental design. C57BL/6J breeder mice were maintained in a 12 h/12 h light/dark (LD) cycle. For mating, male and female breeders at six to 8 weeks of age were shifted to a short day (SD) cycle of 8 h/8 h LD (100 lux) and maintained in the SD cycle throughout gestation. The SD offspring were also kept in the SD cycle until being weaned at postnatal day 21. Control breeders were constantly maintained in the 12 h/12 h LD cycle and control offspring were also weaned at postnatal day 21. Both CTR and SD offspring were maintained in a 12 h/12 h LD cycle unless otherwise mentioned. A battery of behavioral and biochemical tests was performed using Cohort 1 and 2 of CTR and SD offspring at the indicated age. Offspring Cohort 3 was used for wheel-running behavioral tests only.

C57BL/6J breeder mice were purchased from Jackson Laboratory. Animals were housed in the animal facility at the University of Minnesota, Duluth with free access to mouse chow and tap water. The ambient temperature was maintained at 22 ± 1 °C, with humidity levels between 35% and 45%. Mice were maintained in a 12 h/12 h light/dark (LD) cycle. For mating, male and female breeders at 6 weeks of age were shifted to a short day (SD) cycle of 8 h/8 h LD (100 lux). SD offspring were kept in the 8 h/8 h LD cycle until weaning at postnatal day 21 when they were transferred to the 12 h/12 h LD cycle. Control breeders and offspring were constantly maintained in the 12 h/12 h LD cycle (Fig. 7). All tests were performed using 6–8-week-old offspring (at least 5 weeks after weaning). The ratio of male to female mice used was 1:1. All protocols were approved by the Institutional Animal Care and Use Committee at the University of Minnesota. We have complied with all relevant ethical regulations for animal use.

### Mouse behavior assay

After weaning, both the CTR and SD mice were maintained in a standard 12 h/12 h Light/Dark cycle. All behavioral tests were performed in the light phase from ZT2-5 (2–5 h after light-on) under dim red light.

#### Three chamber test

The three-chamber test was conducted using a transparent polyvinyl chloride (PVC) apparatus measuring 60 cm × 40 cm × 20 cm, comprising three compartments, each measuring 20 cm × 40 cm × 20 cm as per previous reports^6,37^. First, a test animal is allowed to freely explore three chambers for 10 min for habituation. Following this, it encounters a stranger mouse in one side compartment while the opposite compartment remains empty. This phase was termed as the sociability phase that lasted for 5 min. Subsequently, a new stranger mouse is introduced into the empty compartment, and the mouse is given another 5 min to explore, known as the social novelty phase. Mouse movements and interactions are meticulously recorded using the ANY-maze software from Stoelting Co (Wood Dale, IL, USA). This comprehensive analysis determined the duration of time spent in each chamber, the number of entries into each chamber, and the duration of time spent in sniffing behavior, defined as the test mouse bringing its nose within 2 cm of the cup or placing its forelimbs on the cup.

#### Marble burying test

This test was performed to check repetitive behavior as reported^38^. In this test, a standard mouse cage was prepared with a 5 cm layer of corncob bedding, ensuring it was evenly compressed for a flat surface. A total of 15 marbles were arranged in three rows of five, spaced 4 cm apart, on the bedding. Then, a test animal was placed in one corner of the cage and allocated to explore for 30 min. Following the session, the number of marbles meeting the “buried” criterion (at least 2/3 covered by bedding) was recorded.

#### Mouse grooming behaviors

To evaluate repetitive and stereotypical behaviors in mice, we conducted a mouse grooming analysis^37^. Mice were given 30 min to acclimate in a standard cage. Following this, they were recorded for 10 min to observe spontaneous self-grooming across all body regions. To examine induced grooming, mice received a single water puff to mist their face and head, followed by a 10-min video recording period to monitor induced grooming behaviors. Videos were carefully reviewed by blind researchers, who recorded the number of grooming bouts and the duration of each grooming episode. Grooming was defined as either a stroke of the forepaws across the head and face or body licking.

#### Novel object recognition (NOR) test

This test was performed as per the protocol described by the Vogel-Ciernia and Wood^39^. This test started with a 10-min habituation phase where the mouse explored a 40 × 40 × 30 cm open field arena devoid of objects, one day prior to the training. On the training day, two glass bottles were positioned at opposing corners of the open arena and the mouse was allowed to freely explore for 10 min, after which it was returned to its cage. After a 24-h period, one of the glass bottles was replaced with a wooden cube, serving as the novel object. The mouse was then reintroduced to the arena, and its behavior was recorded for a 5-min duration. The time spent investigating both the familiar glass bottle and the novel wooden cube was documented using a high-resolution camera and subsequently analyzed through the ANY-maze system. The key metrics assessed in this test included the time spent with the novel (N) and familiar (F) objects, the total distance covered, and the Discrimination Index (DI) calculated as (Time_novel_−Time_familiar_)/(Time_novel_ + Time_familiar_).

#### Object location memory (OLM) test

This test was performed as published^26,39^. The OLM test commenced with a 10-min habituation phase wherein the mouse explored a 40 × 40 × 30 cm open field arena devoid of objects, a day prior to training. On the training day, the mouse encountered two identical glass bottles strategically positioned at opposing corners of the open field arena, each situated 5 cm from the walls. The mouse was given a 10-min window to freely explore both the arena and the objects before returning to its home cage. Following another 24-h interval, one of the bottles remained in its original position (familiar object), while the other was relocated adjacent to a corner (novel object). The mouse was then reintroduced to the arena for a 5-min exploration session, during which its behavior was recorded via video documentation. The ANY-maze mouse tracking system was employed for activity analysis, recording the time spent by the mouse exploring the novel (N) and familiar (F) objects, total distance traveled. Additionally, the discrimination index (DI) was calculated using the formula (Time_novel_−Time_familiar_)/(Time_novel_ + Time_familiar_), providing a measure of the mouse’s preference for the novel object over the familiar one.

#### Barnes maze test

The Barnes maze test was performed as reported^40,41^. It employs a circular platform with a diameter of 92 cm and is raised to a height of 95 cm. It features 20 evenly spaced apertures, each with a diameter of 5 cm, positioned 2 cm from the edge. Beneath one of these apertures lies an escape box measuring 20 × 9 × 9 cm, facilitating the mouse’s escape. The test regimen initiates with a habituation phase, during which mice undergo two stages: first, they are gently placed in the escape box for a period of 2 min, followed by a guided introduction to the escape box, where they remain for an additional 2 min. In the subsequent 4 days, constituting the acquisition phase, mice receive 1 trial per day. Each trial commences with the mouse briefly situated in a 10 cm high cylindrical black start chamber at the maze’s center, with an allotted 5-min span to locate the escape box. In instances where a mouse fails to locate the escape box within the designated timeframe, it is gently guided to the box and allowed to stay inside for 2 min before being returned to its home cage. The entire session was captured on video, and subsequent analysis was conducted using the ANY-maze software. Specifically, the duration taken to find and enter the escape box was precisely recorded. After 24 h of the training period, a probe test was executed, this time with the escape box removed. Mice were granted 90 s to explore the maze, and their time allocation in each of the four quadrants, coupled with travel distances, were carefully assessed. This thorough protocol affords a comprehensive evaluation of spatial memory and learning aptitude in mice.

#### Open field test (OFT)

The open field test was utilized to evaluate anxiety-related behaviors in mice^37^. This test involved placing the mouse in a PVC arena measuring 40 × 40 × 30 cm, allowing them to freely explore for a duration of 5 min. Video recording captured their movements, subsequently analyzed via the ANY-maze video tracking system to determine parameters like total travel distance, distance traveled and time allocation in specific zones (center and outside). The center zone, a 20 × 20 cm square at the arena’s midpoint, and the outer zone, comprising the remainder of the arena, were defined for analysis.

#### Elevated plus maze (EPM)

This was performed as reported^42^. This test involved employing an apparatus comprising two open arms (50 cm in length, 10 cm in width) and two closed arms of identical dimensions but with 15 cm high walls, arranged perpendicularly. Positioned 50 cm above the floor, the maze induces a level of anxiety in rodents due to their natural aversion to elevated and exposed environments. A mouse, initially placed at the center of the maze facing an open arm, was allowed to freely explore for a 5-min duration. The experiment was meticulously recorded through video documentation and subsequently analyzed utilizing the ANY-maze animal behavior tracking system (Stoelting Co., IL), which facilitated the measurement of time spent by the mice in both open and closed arms.

#### Rotarod test

The experiment utilized a rotarod apparatus (Rotamex-5, Columbus Instruments, Columbus, OH, USA) featuring a rotating cylinder for mice that measured 3 × 9.5 cm and operated at speeds ranging from 4 to 40 rpm. Initially, mice underwent pretraining, where they were required to remain on the cylinder at the minimum speed of 4 rpm for at least 1 min. During the training phase, mice were placed on the rotarod, which accelerated from 4 to 40 rpm over 5 min (acceleration rate: 7.2 rpm/min). The time taken for the mice to fall and the speed at which they fell were recorded. Training consisted of eight sessions spread over three consecutive days: three sessions each day on days 1 and 2 and two sessions on day 3, with a 10-min break between each session.

#### Test suspension test (TST)

The tail suspension performed as per published^43^. This test began with a habituation period for mice. Subsequently, the mice were visually isolated from their immediate surroundings, and their tails were securely fastened to a device positioned 50 cm above the floor using tape. The ensuing suspension phase was recorded via video for a duration of 5 min. Following the test, the tape securing the tail was carefully removed, and the mice were then returned to their respective home cages. Importantly, all experiments were video-recorded and analyzed by impartial, blinded researchers. The immobility time indicates the time the mice remained motionless during the final 4 min of the test. Additionally, latency, representing the duration from the initiation of the test to the first occurrence of immobility, was recorded.

#### Forced swim test (FST)

The forced swim test was performed as published^43^. It proceeded as follows: after an initial habituation period, individual mice were placed into a glass cylinder with a diameter of 20 cm, filled with 15 cm of water held at a controlled temperature of 25 ± 1 °C. Each mouse was subjected to a 6-min immersion. After the test, the mice were gently dried with clean towels and then returned to their respective home cages. All experimental sessions were thoroughly video-recorded and later analyzed by unbiased researchers. The immobility time represents the time the mice exhibited immobility during the last 4 min of the test. Furthermore, latency, signifying the duration from the test’s onset to the initial immobile episode, was recorded.

#### Novelty-suppressed feeding (NSF)

In accordance with the methodology outlined by Ramaker and Dulawa^44^, the novelty-suppressed feeding test was conducted as follows: each mouse, having undergone a 24-h food deprivation period prior to testing, was introduced into a 40 cm × 40 cm open field arena, with a food pellet positioned at the center. Video recordings of the experiments were subsequently analyzed by researchers who were blinded to the experimental conditions. The latency to initiate feeding, defined as the duration taken to make the first bite of food, was recorded. Following the test, the mice were gently returned to their respective home cages. Moreover, the amount of food consumed during the initial 5 min for each mouse was quantified, facilitating the identification and exclusion of any potential feeding-related issues or deficiencies in these animals.

#### Circadian behavioral assay

After weaning from their respective LD cycles, CTR and SD mice were transferred to an 8 h/8 h Light/Dark (8 h/8 h LD) cycle, and their wheel-running activities were recorded. After 28 d in 8 h/8 h LD, mice were transferred to constant darkness (DD) and kept in DD for 24 d. Mice were individually housed in cages equipped with running wheels and wheel-running activities were recorded by the ClockLab software (Actimetrics, Wilmette, USA). Data were analyzed using the ClockLab Analysis software. The experimenter was blinded to the groups of the animals.

#### Western blotting

The western blotting commenced with the homogenization of forebrain tissue using a pestle grinder from Fisher Scientific Limited, Nepean, ON, Canada, followed by treatment with a lysis buffer. Adhering to the outlined methodology, protein extraction products were subsequently applied onto a 10% SDS–PAGE gel for electrophoresis. Next, the blots underwent transfer onto 0.45 μm polyvinylidene difluoride membranes (Immobilon-P, Merck Millipore Ltd., Carrigtwohill, Ireland). Following blocking with 10% fat-free milk from Fisher Scientific, Fair Lawn, NJ, USA, the membranes were immersed in PBST (PBS with 1% Triton X-100) supplemented with 5% BSA, alongside primary antibodies directed against p-S6 (1:1000, CST 2215, Cell Signaling Technology, Danvers, MA), S6 (1:1000, SC-74459, Santa Cruz Biotechnology, Dallas, TX, USA), p-S6K1 (1:1000, CST 9206, Cell Signaling Technology, Danvers, MA), S6K1 (1:1000, CST 34475, Cell Signaling Technology, Danvers, MA), p-ERK (1:1000, CST 4370, Cell Signaling Technology, Danvers, MA), ERK (1:1000, CST 4695, Cell Signaling Technology, Danvers, MA), p-eIF4E (1:2000, 44-528G, Invitrogen, Waltham, MA), eIF4E (1:1000, BD-610270, BD, Franklin Lakes, NJ) and β-actin (1:5000, A5441, Sigma-Aldrich, St. Louis, MO) overnight at 4 °C. Following that, the membranes were immersed in PBST with 5% fat-free milk and were exposed to an HRP-conjugated secondary antibody (1:5000 dilution; donkey anti-rabbit: NA934V; sheep anti-mouse: NA931V, GE Healthcare, Piscataway, NJ). The membranes underwent at least three wash cycles (5 min each) using PBST between each antibody treatment. Chemiluminescence was induced using Western Lightning Chemiluminescence Reagents (PerkinElmer, Inc., Waltham, MA, USA) and captured using X-ray films. The prestained protein ladders remained visible on the blotted membrane. The X-ray films were overlaid onto the blotted membranes, and the positions of the protein ladder bands were marked manually on the X-ray films. X-ray films were scanned, images were digitized, and blot densities were quantified using Image-J software (National Institutes of Health).

#### Immunostaining and imaging data analysis

For immunohistochemistry, the brain sections of 40 µm thickness were first incubated in PBS with 20% methanol and 0.3% H_2_O_2_ for 30 min and then blocked in PBS with 10% goat serum for 1 h at room temperature. Sections were incubated in PBS with the primary antibody, rabbit anti-pS6 (1:3000, CST 2215, Cell Signaling Technology, Danvers, MA) overnight at 4 °C. On the following day, sections were incubated in PBS with a biotinylated anti-rabbit IgG secondary antibody (1:400, Vector Laboratories, Burlingame, CA) for 2 h at room temperature. Next, sections were incubated in PBS with the avidin/biotin HRP complex (Vector Laboratories, Newark, CA, USA) for 1 h at room temperature. The signal was visualized using a nickel-enhanced DAB substrate, following the instructions provided by the manufacturer (Vector Laboratories, Newark, CA, USA). Between each step, slices were washed three times for 5 min with PBS. Finally, sections were mounted on slides and covered using a Permount mounting medium (Fisher Scientific, Houston, TX).

Bright-field images were acquired using a DMi8 inverted Leica microscope equipped with an integrated digital camera (Leica, Wetzlar, Germany). All microscope settings, including light intensity, exposure time, and contrast, were kept constant across all datasets. For the quantification of staining density, images of different brain regions were captured at ×1.25 magnification and converted to 8-bit grayscale format. Specific regions within the brain slice were selected, and their intensities were measured using Adobe Photoshop software (Adobe Systems Incorporated, San Jose, CA). Background intensity was also recorded for each image, and the normalized protein intensity was calculated by subtracting the background intensity from the measured values. The relative intensities of each region were standardized as ratios relative to the intensities of the control.

#### Statistical analysis and reproducibility

Statistical analysis and graph plotting were conducted using GraphPad Prism 8 (GraphPad Software, La Jolla, CA, USA). The data were presented as individual values and as mean ± standard error of the mean (SEM). A *p*-value of <0.05 was deemed statistically significant. Detailed statistical methods and results are provided in the figure legends. While no statistical methods were employed to predetermine the sample sizes, our sample sizes were comparable to those reported in prior studies.

### Reporting summary

Further information on research design is available in the Nature Portfolio Reporting Summary linked to this article.

## Supplementary information

Supplementary Information

Description of Additional Supplementary Files

Supplementary Data 1

Reporting Summary

## Data Availability

The numerical source data for graphs and charts are provided in Supplementary Data 1. All other data are available from the corresponding author upon reasonable request.
