# Supplementary material for: Circadian desynchrony in early life leads to enduring autistic-like behavioral changes in adulthood
Source: Commun Biol. 2024 Nov 11;7:1485. doi: 10.1038/s42003-024-07131-3 (PMC11555041; doi:10.1038/s42003-024-07131-3)
Supplement: Supplementary file 1 — Supplementary Information [file 42003_2024_7131_MOESM1_ESM.pdf]

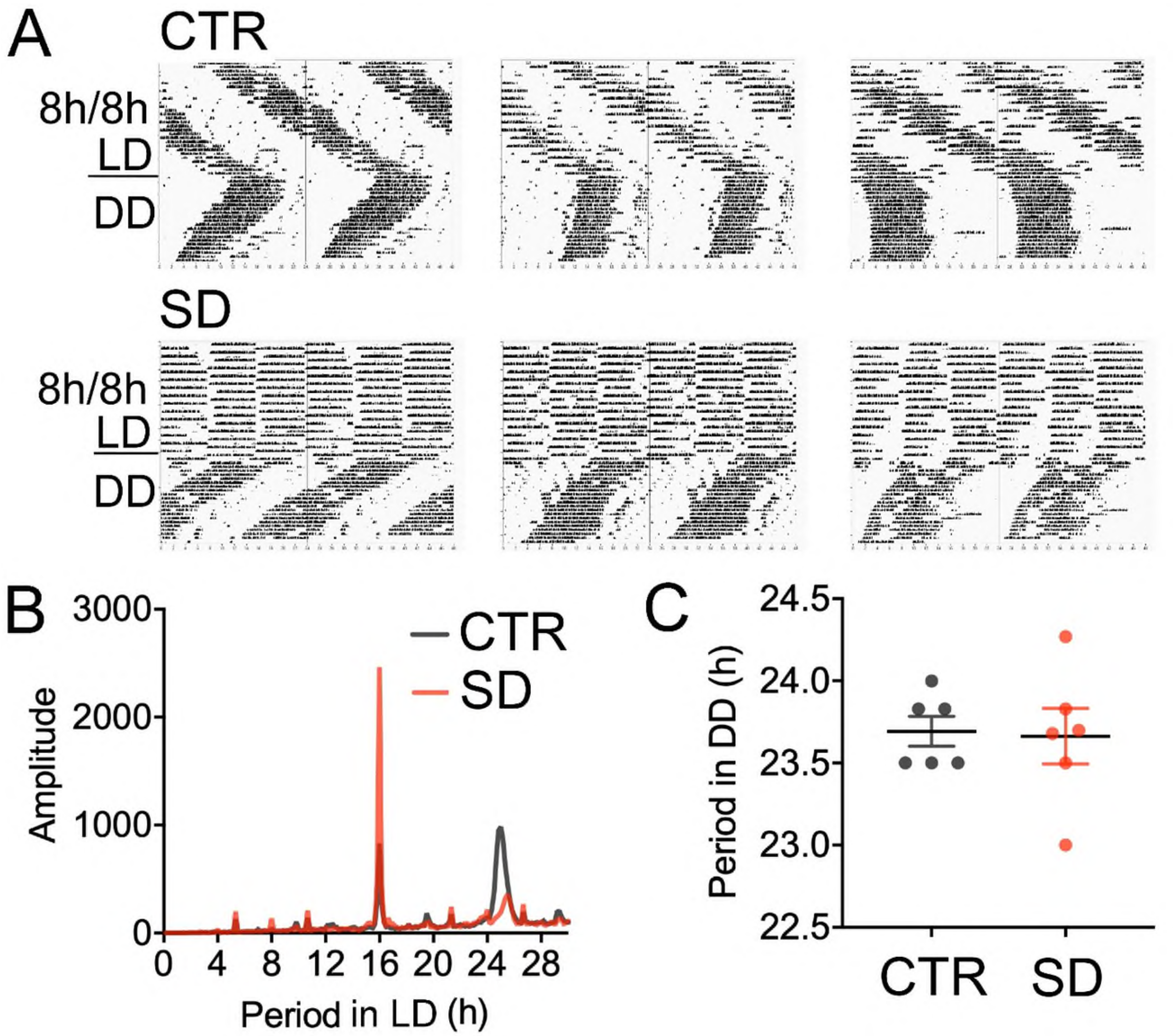

Figure S1. Wheel-running behavior in CTR and SD mice. After weaning from their respective LD cycles, CTR and SD mice were transferred to an 8h/8h Light/Dark (8h/8h LD) cycle and their wheel-running activities were recorded. After 28 d in 8h/8h LD, mice were transferred to constant darkness (DD) and kept in DD for 24 d. (A) Wheel-running actograms in 8h/8h LD and DD. Three representative actograms are shown from each group. (B) Periodograms of CTR and SD mice in 8h/8h LD. Note that the SD mice entrained to the 8h/8h LD cycle and showed a period of 16 h, while the CTR mice failed to entrain to the 8h/8h LD cycle and showed a free-running period of ~25 h. (C) Period length of CTR and SD mice in DD. Note that both the CTR and SD mice exhibited a circadian period of ~23.7 h and there is no significant difference between the two groups (n=6 for each group. $t = 0.1561$ $p = 0.8790$, via Student's t test).

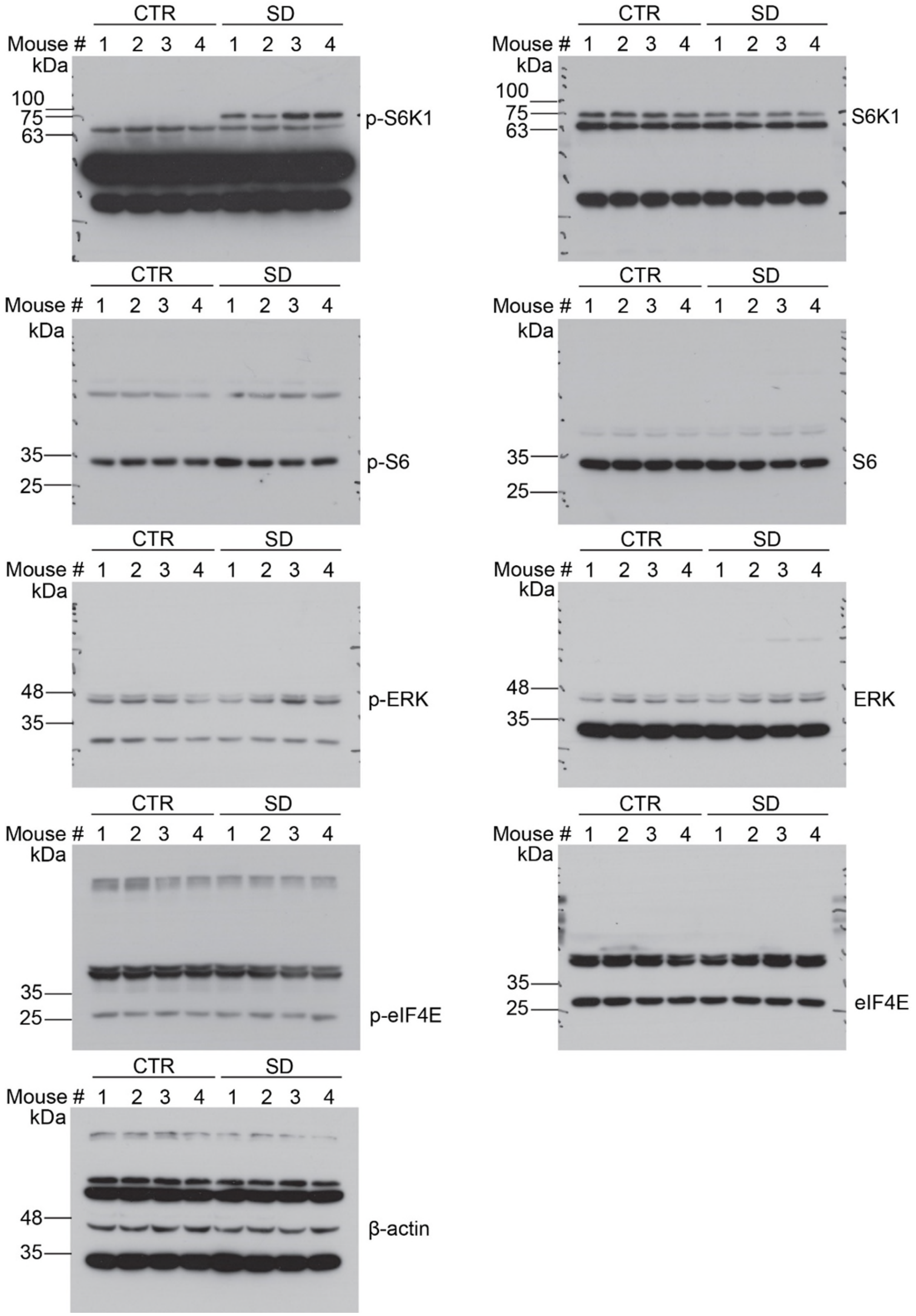

Figure S2. Uncropped western blotting images (related to Figure 7).
