# Supplementary material for: Circadian desynchrony in early life leads to enduring autistic-like behavioral changes in adulthood
Source: Commun Biol. 2024 Nov 11;7:1485. doi: 10.1038/s42003-024-07131-3 (PMC11555041; doi:10.1038/s42003-024-07131-3)
Supplement: Supplementary file 4 — Reporting Summary [file 42003_2024_7131_MOESM4_ESM.pdf]

nature portfolio

Corresponding author(s): Ruifeng Cao

Last updated by author(s): Jul 30, 2024

Reporting Summary

Nature Portfolio wishes to improve the reproducibility of the work that we publish. This form provides structure for consistency and transparency in reporting. For further information on Nature Portfolio policies, see our Editorial Policies and the Editorial Policy Checklist.

Statistics

For all statistical analyses, confirm that the following items are present in the figure legend, table legend, main text, or Methods section.

n/a	Confirmed	
☐	☒	The exact sample size (*n*) for each experimental group/condition, given as a discrete number and unit of measurement
☐	☒	A statement on whether measurements were taken from distinct samples or whether the same sample was measured repeatedly
☒	☐	The statistical test(s) used AND whether they are one- or two-sided<br>*Only common tests should be described solely by name; describe more complex techniques in the Methods section.*
☐	☒	A description of all covariates tested
☐	☒	A description of any assumptions or corrections, such as tests of normality and adjustment for multiple comparisons
☐	☒	A full description of the statistical parameters including central tendency (e.g. means) or other basic estimates (e.g. regression coefficient) AND variation (e.g. standard deviation) or associated estimates of uncertainty (e.g. confidence intervals)
☐	☒	For null hypothesis testing, the test statistic (e.g. *F*, *t*, *r*) with confidence intervals, effect sizes, degrees of freedom and *P* value noted<br>*Give P values as exact values whenever suitable.*
☒	☐	For Bayesian analysis, information on the choice of priors and Markov chain Monte Carlo settings
☒	☐	For hierarchical and complex designs, identification of the appropriate level for tests and full reporting of outcomes
☒	☐	Estimates of effect sizes (e.g. Cohen's *d*, Pearson's *r*), indicating how they were calculated

Our web collection on statistics for biologists contains articles on many of the points above.

Software and code

Policy information about availability of computer code

Data collection	N/A
Data analysis	Statistical analysis and graph plotting were conducted using GraphPad Prism 8 (GraphPad Software, La Jolla, CA, USA)

For manuscripts utilizing custom algorithms or software that are central to the research but not yet described in published literature, software must be made available to editors and reviewers. We strongly encourage code deposition in a community repository (e.g. GitHub). See the Nature Portfolio guidelines for submitting code & software for further information.

Data

Policy information about availability of data

All manuscripts must include a data availability statement. This statement should provide the following information, where applicable:

- Accession codes, unique identifiers, or web links for publicly available datasets
- A description of any restrictions on data availability
- For clinical datasets or third party data, please ensure that the statement adheres to our policy

Data availability
All data associated with this study are present in the article. The raw data are available from the corresponding author on reasonable request.

Research involving human participants, their data, or biological material

Policy information about studies with human participants or human data. See also policy information about sex, gender (identity/presentation), and sexual orientation and race, ethnicity and racism.

Reporting on sex and gender	N/A
Reporting on race, ethnicity, or other socially relevant groupings	N/A
Population characteristics	N/A
Recruitment	N/A
Ethics oversight	N/A

Note that full information on the approval of the study protocol must also be provided in the manuscript.

Field-specific reporting

Please select the one below that is the best fit for your research. If you are not sure, read the appropriate sections before making your selection.

☒ Life sciences ☐ Behavioural & social sciences ☐ Ecological, evolutionary & environmental sciences

For a reference copy of the document with all sections, see nature.com/documents/nr-reporting-summary-flat.pdf

Life sciences study design

All studies must disclose on these points even when the disclosure is negative.

Sample size	While no statistical methods were employed to predetermine the sample sizes, our sample sizes were comparable to those reported in prior studies.
Data exclusions	All data are included except one control mouse was identified as an outlier and excluded for NOR, OLM and Barnes Maze test.
Replication	Each test was performed in multiple cohorts of mice and the results are compared between cohorts to ensure reproducibility..
Randomization	C57BL/6J mice were randomly assigned as the breeders of CTR or SD mice.
Blinding	The experimenter was blinded to the groups of the animals.

Reporting for specific materials, systems and methods

We require information from authors about some types of materials, experimental systems and methods used in many studies. Here, indicate whether each material, system or method listed is relevant to your study. If you are not sure if a list item applies to your research, read the appropriate section before selecting a response.

Materials & experimental systems

n/a	Involved in the study	
☐	☒	Antibodies
☒	☐	Eukaryotic cell lines
☒	☐	Palaeontology and archaeology
☐	☒	Animals and other organisms
☒	☐	Clinical data
☒	☐	Dual use research of concern
☒	☐	Plants

Methods

n/a	Involved in the study	
☒	☐	ChIP-seq
☒	☐	Flow cytometry
☒	☐	MRI-based neuroimaging

Antibodies

Antibodies used	p-S6 (1:1000, CST 2215, Cell Signaling Technology, Danvers, MA), S6 (1:1000, SC-74459, Santa Cruz Biotechnology, Dallas, TX, USA), p-S6K1 (1:1000, CST 9206, Cell Signaling Technology, Danvers, MA), S6K1 (1:1000, CST 34475, Cell Signaling Technology, Danvers, MA), p-ERK (1:1000, CST 4370, Cell Signaling Technology, Danvers, MA), ERK (1:1000, CST 4695, Cell Signaling Technology, Danvers, MA),

	p-eIF4E (1:2000, 44-528G, Invitrogen, Waltham, MA), eIF4E (1:1000, BD-610270, BD, Franklin Lakes, NJ) and β-actin (1:5000, A5441, Sigma-Aldrich, St. Louis, MO), donkey anti-rabbit: NA934V; sheep anti-mouse: NA931V (GE Healthcare, Piscataway, NJ).
Validation	All antibodies have been validated in previous publications.

Animals and other research organisms

Policy information about studies involving animals; ARRIVE guidelines recommended for reporting animal research, and Sex and Gender in Research

Laboratory animals	C57BL/6J
Wild animals	N/A
Reporting on sex	Both sexes were used as a 1:1 ratio.
Field-collected samples	N/A
Ethics oversight	All protocols were approved by the Institutional Animal Care and Use Committee at the University of Minnesota.

Note that full information on the approval of the study protocol must also be provided in the manuscript.

Plants

Seed stocks	N/A
Novel plant genotypes	N/A
Authentication	N/A
